# Interleukin-24 Immunobiology and Its Roles in Inflammatory Diseases

**DOI:** 10.3390/ijms23020627

**Published:** 2022-01-06

**Authors:** Yajie Zhong, Xuan Zhang, Waipo Chong

**Affiliations:** State Key Laboratory of Ophthalmology, Zhongshan Ophthalmic Center, Sun Yat-Sen University, Guangzhou 510060, China; zhongyj27@mail2.sysu.edu.cn (Y.Z.); zhangx726@mail2.sysu.edu.cn (X.Z.)

**Keywords:** IL-24, inflammation, autoimmune disease

## Abstract

Interleukin (IL)-24 belongs to the IL-10 family and signals through two receptor complexes, i.e., IL-20RA/IL-20RB and IL-20RB/IL22RA1. It is a multifunctional cytokine that can regulate immune response, tissue homeostasis, host defense, and oncogenesis. Elevation of IL-24 is associated with chronic inflammation and autoimmune diseases, such as psoriasis, rheumatoid arthritis (RA), and inflammatory bowel disease (IBD). Its pathogenicity has been confirmed by inducing inflammation and immune cell infiltration for tissue damage. However, recent studies also revealed their suppressive functions in regulating immune cells, including T cells, B cells, natural killer (NK) cells, and macrophages. The tolerogenic properties of IL-24 were reported in various animal models of autoimmune diseases, suggesting the complex functions of IL-24 in regulating autoimmunity. In this review, we discuss the immunoregulatory functions of IL-24 and its roles in autoimmune diseases.

## 1. Introduction

Interleukin (IL)-24 is a member of the IL-10 family of cytokines, which also contains IL-10, IL-19, IL-20, IL-22, IL-26, and three members from the type III interferons, i.e., IL-28A, IL28B, and IL-29. The IL-24 gene is located in the human chromosome 1q32, with seven exons and six introns. This region is considered the “IL-10 family gene cluster” as it also contains the IL-10, IL-19, and IL-20 genes. The open reading frame of the IL-24 gene consists of 206 amino acid residues, with 51 of them serving as a signal peptide [1]. Mature IL-24 consists of 155 amino acid residues with a molecular mass ranging from 18.3 kDa to 35 kDa, depending on differences in N-lined glycosylation [2]. IL-24 is broadly expressed by both immune cells [3] and non-immune cells [3,4,5,6,7] (summarized in Table 1), suggesting that it acts as a multifunctional cytokine to regulate a wide variety of different cell types. Splice variants of IL-24 with distinct biological functions have also been reported [7].

**Figure 1:**
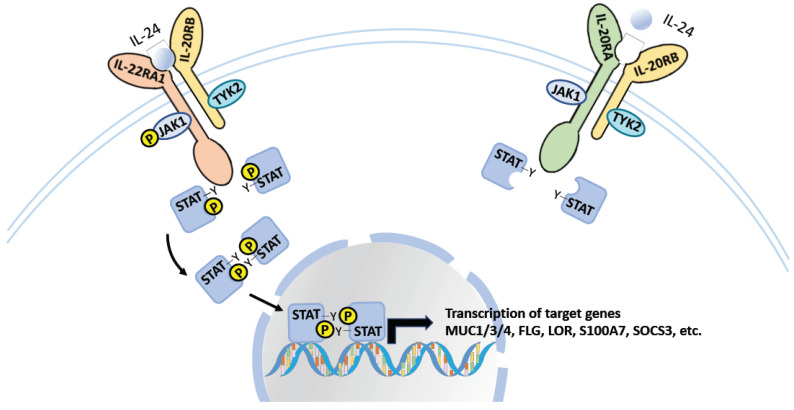
The IL-24 signaling. IL-24 signals through the heterodimeric receptor, IL-20RA/IL-20RB and IL-22RA1/IL-20RB. Both receptors signal through the STAT-JAK signaling pathway for the regulation of gene expression.

IL-24, together with IL-19 and IL-20, are considered IL-20 receptor cytokines because they signal through the same IL-20 receptor β-subunit (IL-20RB), which can form a heterodimeric receptor with either the IL-20 receptor α-subunit (IL-20RA) or the IL-22 receptor α1-subunit (IL-22RA1)—known as the type I and type II complex, respectively (Figure 1) [7]. While IL-19 is only recognized by the IL-20 receptor type I complex, IL-20 and IL-24 signal through both receptor complexes. It is not yet clear whether the binding of IL-20 or IL-24 to these two receptor complexes triggers different signaling pathways and cellular responses, which future comprehensive studies may elucidate.

IL-24 signaling is mediated through the Janus kinase (JAK)-signal transducer and the activator of transcription (STAT) pathways—involving STAT1 and STAT3 [19]—and has diverse impacts on cell differentiation, proliferation, and apoptosis (Figure 1). Because of the high expression of these receptors in epithelial cells, the role of IL-24 in tissue inflammation and autoimmune diseases has been studied intensively. In addition, recent studies demonstrated the capacity of IL-24 to regulate different immune cells, including CD4^+^ and CD8^+^ T cells [8,20], B cells [15], natural killer (NK) cells [21], and macrophages [22]. However, despite the recent evidence supporting the role of IL-24 in the regulation of different types of immune cells, its mechanism and involvement in human diseases has yet to be clearly characterized. In this review, we highlight the recent findings regarding the function of IL-24 in the regulation of immune cells and its role in autoimmune diseases.

## 2. Immunobiology of IL-24

IL-24 was discovered by subtractive hybridization from growth-arrested and terminally differentiated human melanoma cells, and was known as melanoma-differentiation-associated gene 7 (MAD-7) [23]. Subsequent studies further confirmed its function as a tumor suppressor gene in various tumor models due to its ability to arrest tumor cell growth and induce apoptosis and autophagy [24]. These include models of breast cancer [25], prostate cancer [26], lung cancer [27], liver cancer [28], melanoma [29], leukemia [30], and others [24]. Interestingly, instead of the more commonly seen JAK/STAT signaling pathway, these tumor suppressive effects can also be mediated via activation of the p38 mitogen-activated protein kinase (MAPK) pathway [29,31,32], suggesting the complexity of IL-24 signaling in the regulation of cellular responses. Later studies demonstrated that IL-24 can exert proinflammatory effects and is associated with susceptibility to a number of autoimmune diseases, such as psoriasis and rheumatoid arthritis (RA) [7]. However, we and others recently revealed the tolerogenic properties of IL-24 to inhibit the effector functions of immune cells, suggesting that IL-24 is a multifunctional cytokine with both pathogenic and tolerogenic properties. In this section, we discuss the role of IL-24 in the regulation of various immune cells, which may be implicated in the pathogenesis of inflammatory and autoimmune diseases.

### 2.1. T Cells

CD4^+^ helper T cells and CD8^+^ cytotoxic T cells represent the most dominant effector cells in adaptive immunity. Loss of T cell tolerance is one of the major causes of tissue inflammation and autoimmune diseases. IL-24 can be produced by activated T cells—particularly T helper (Th) 2 cells—and was shown to be capable of regulating their effector functions. Schaefer et al. reported the expression of IL-24 from Th2 cells, which required the activation of protein kinase C through TCR signaling and STAT6 through IL4 receptor signaling [9]. A follow-up mechanistic study determined that the binding of STAT6 to the IL-24 promoter is facilitated by its interaction with c-Jun—an important complement of the activator protein-1 (AP-1) complex—for the optimal expression of IL-24 in Th2 cells [10]. In accordance, a chromatin immunoprecipitation-sequencing (ChIP-seq) study also confirmed activation of the IL-24 gene by STAT6 in Th2 cells [11]. Therefore, together with the other two IL-20 receptor cytokines, IL-19 and IL-20, IL-24 is considered a Th2 cytokine. However, unlike IL-19 and IL-20, IL-24 cannot promote Th2 differentiation from naive CD4^+^ T cells [33] suggesting that, although they share structural homology as well as receptors and downstream signaling pathways, they have distinct biological functions in the regulation of T cell responses.

As one of the Th2 cytokines, IL-24 displays suppressive functions against other T cell lineages, including the interferon (IFN)-γ producing Th1/Tc1 and IL-17A-producing Th17/Tc17 cells. Anuradha et al. reported increased expression of IL-24 by CD4^+^ and CD8^+^ T cells in patients with lymphatic filariasis [12], and found that neutralization of IL-24 from filarial antigen-stimulated whole blood cells of these patients significantly increased the number of IFNγ–producing Th1/Tc1 and IL17A-producing Th17/Tc17 cells. Kumar et al. reported a very similar observation in patients with pulmonary tuberculosis, whose T cells expressed elevated levels of IL-24, and IL-24 limited the expression of IFN-γ and IL-17A from Th1/Tc1 and Th17/Tc17 cells, respectively [13]. In another study using naive CD4^+^ T cells from healthy individuals, Oral et al. demonstrated that IL-24 inhibited IFN-γ production under anti-CD3/CD28 antibody activation [33]. In contrast to these observations, IL-24 was found to promote the activation of CD4^+^ T cells and CD8^+^ T cells isolated from colorectal adenocarcinoma with elevated IFN-γ and IL-17A expression [34]. Further investigation is necessary to clarify the effects of IL-24 in regulating the cytokine profile of T cells from different microenvironments.

The Th17 cell has been studied intensively in autoimmune diseases due to its capacity to induce inflammation. Its signature cytokines, including IL-17A and IL-17F, promote the production of proinflammatory molecules from various immune and non-immune cells, and recruit inflammatory cells—such as neutrophils and macrophages—to the inflammatory site [35]. Recent studies revealed that Th17 cells also express IL-24. An RNAseq-based analysis of Th17 cells reported the gene expression of IL-24 in the late-differentiation phase [14]. Subsequently, our group confirmed the expression of IL-24 by induced Th17 cells in vitro, as well as uveitogenic Th17 cells from the animal model of uveitis, experimental autoimmune uveitis (EAU) [8]. In addition, we found that IL-24 negatively feedbacks with Th17 cells for suppression of the Th17 effector cytokines, including IL-17F and GM-CSF, and hence, resolves the ocular inflammation observed in Th17-mediated EAU [8]. Interestingly, our mechanistic study revealed that IL-24 expression in Th17 cells is induced by its own IL-17A through nuclear factor kappa-light-chain-enhancer of activated B cells (NFκB) signaling in an autocrine manner [8]. Taken together, IL-24 in differentiated Th17 cells has a role in regulating the pathogenic Th17 response, and may participate in the resolution of tissue inflammation and autoimmune diseases.

### 2.2. B Cells

B cells are another arm of adaptive immunity. They produce antibodies and immunoregulatory molecules for tissue inflammation. They are important for the structure of lymphoid tissue and regulate T cell activation through antigen presentation and co-stimulation. Maarof et al. reported that follicular B cells—predominantly in CD27^+^ memory B cells and CD5^+^ B cells—express high levels of IL-24 upon BCR activation and CD40–CD40L ligation [15]. In vitro experiments demonstrated that IL-24 promotes CD40L-induced B cell proliferation, but inhibits the formation of plasma cells, Immunoglobulin (Ig) G production, and IL-10 expression [15]. However, another study reported that IL-24 mediates B cell apoptosis by inducing the genes of the mitochondrial apoptotic pathway during the late phase of B cell differentiation, and inhibiting genes that are involved in DNA replication and metabolism in the early phase [36]. Further studies are required to characterize the role of IL-24 in regulating B cells.

### 2.3. NK Cells

NK cells are the third largest lymphocyte population and play an important role in innate immunity by eliminating transformed cells and virus-infected cells. Recent studies, including ours, confirmed the role of NK cells in the regulation of adaptive immunity through expression of immunoregulatory cytokines, including IFN-γ [37,38]. Human NK cells also express IL-24 with mitogen stimulation [16]. Type 1 IFNs were also demonstrated to induce IL-24 production in murine NK cells in a STAT6-dependent manner [17]. Although IL-24 is not capable of activating peripheral NK cells [39], Yang et al. demonstrated that decidual stromal cells (DSCs) produce IL-24 to promote the differentiation of CD56^bright^CD16^−^ NK cells with regulatory phenotype that express high levels of the inhibitory receptors killer-cell immunoglobulin-like receptor (KIR)2DL1 and KIR3DL1, and inhibitory cytokines including transforming growth factor (TGF)-β, IL-8, and IL-10 [21].

### 2.4. Macrophages

Macrophages are phagocytes that help to remove pathogens, dead and transformed cells, and foreign substances. They serve as the effector cells in innate immunity, but are also involved in regulating adaptive immunity due to their capacity to present antigens to both T and B cells. They modulate inflammation by producing both pro- and anti-inflammatory cytokines. Upon LPS and IL-4 stimulation, macrophages express high levels of IL-24, which require STAT6 activation [17,18]. IL-24 was shown to synergize with IL-4 in the induction of anti-inflammatory M2 macrophages by inhibiting suppressor of cytokine signaling 1 (SOCS1) and SOCS3, and promoting the STAT6/PPARγ signaling pathways [22]. In addition, IL-24 from DSCs induces both apoptosis and proliferation of decidual macrophages, suggesting its role in promoting the renewal of these cells [40]. Interestingly, IL-24 can also induce human monocytes migration in vitro and recruits CD11b^+^ myeloid cells in vivo [41], suggesting its capacity to regulate the immune response as a chemoattractant.

## 3. IL-24 in Inflammatory and Autoimmune Diseases

Inflammatory diseases refer to conditions wherein the immune system attacks the host’s own tissue. Autoimmune diseases involve a loss of self-tolerance that leads to the activation of autoreactive T and/or B cells for tissue inflammation. Upregulation of IL-24 has been associated with many of these diseases (Table 2), and as such it is considered a proinflammatory cytokine. However, recent studies determined that IL-24 has suppressive functions that may display anti-inflammatory properties in some of these inflammatory diseases (Table 2).

### 3.1. Psoriasis

Psoriasis is a chronic inflammatory disease of the skin resulting from the excessive proliferation and differentiation of keratinocytes. IL-20 receptor cytokines, including IL-24, are elevated in the skin lesions of patients with psoriasis [42,43], and genetic overexpression of these cytokines leads to the development of paresis-like disease in mice [3]. In vivo studies revealed that tumor necrosis factor (TNF)-α induces IL-24 to drive psoriasis-like skin inflammation in mice [44]. IL-24 also stimulates the expression of pro-inflammatory molecules from human keratinocytes, including psoriasin, LCN2, IL-20, CXCL1, CXCL8, and CCL20 [44]. IL-17A was identified as another pathogenic cytokine in psoriasis [45]. The IL-23/IL-17 cytokine axis plays an important role in disease pathogenesis [46]. Targeting of IL-17A and/or its receptor is effective in treating psoriasis [47], and administration of secukinumab resolves plaque histopathology and reduces proinflammatory gene expression in patients with psoriasis [48]. Xu et al. recently reported that IL-17A directly induces IL-24 expression in skin fibroblasts and keratinocytes [49]. These results support the pathogenic role of IL-24 in psoriasis and support the hypothesis that its elevated expression may be driven by IL-17A.

### 3.2. Rheumatoid Arthritis

RA is caused by an autoimmune response against the synovial joints. It is characterized by chronic inflammation with immune cell infiltration and synovial cell activation. Although the etiology remains unknown, activation of autoreactive B and T cells was confirmed through the detection of autoantibodies and proinflammatory cytokines. IL-24, together with IL-20, were found to be increased in plasm from patients with RA and spondyloarthropathy [50,51]. In addition, IL-24 was elevated in endothelial and mononuclear cells from synovial membranes of these patients, and stimulated synovial fluid mononuclear cells to express the chemoattractant, CCL2 [50]. Blocking of IL-20 receptor cytokines by IL-20RB-Fc fusion protein was shown to ameliorate the collagen-induced arthritis model in DBA/1 mice [52].

### 3.3. Systemic Lupus Erythematosus

Systemic lupus erythematosus (SLE) is an autoimmune disease that affects many parts of the body, including the skin, kidneys, joints, lungs, and the vascular and nervous systems. It is characterized by the production of autoantibodies—including anti-nuclear (ANAs), anti-double-stranded DNA (dsDNA), anti-Smith antigen (Sm), and anti-ribonucleoproteins (RNP)—against the self-antigen from cell nuclei. IL-24 serum levels are significantly higher in patients with SLE compared with healthy individuals, and elevated IL-24 levels are also associated with disease severity [53]. However, no significant difference in IL-24 serum levels was observed between SLE patients and healthy controls in two other independent studies [39,54]. Future studies are required to investigate the association between IL-24 and SLE.

### 3.4. Inflammatory Bowel Disease

Inflammatory bowel disease (IBD)—including Crohn’s disease (CD) and ulcerative colitis (UC)—can be described as chronic inflammation of the gastrointestinal (GI) tract. Several studies confirmed the expression of IL-24 in inflamed mucosa of patients with IBD [55,56,57]. IL-24 induced SOCS3 and mucins expression in colonic epithelial cells, which can help to protect and maintain epithelial and mucosal integrity [55]. Onody et al. also observed increased expression of IL-24 in the serum and colon samples of children with IBD. IL-24 treatment stimulated HT-29 cells to produce TGF-β and PDGF-B, and CCD-18Co cells to express extracellular matrix (ECM)-related genes, suggesting the potential role of IL-24 in the remodeling of colon tissue [58]. In a follow-up in vivo study, they found that IL-20 receptor cytokine signaling is pathogenic in the dextran sulfate sodium (DSS)-induced colitis model, as *Il20rb* deficiency ameliorates disease development [58]. However, it was noted that *IL20rb* deficiency leads to the loss of not only IL-24-, but also IL-19- and IL-20-mediated signaling; therefore, a more specific study is required to confirm the role of IL-24 in this animal model.

### 3.5. Central Nervous System Autoimmune Diseases

The central nervous system (CNS) evolved to limit inflammation in order to protect delicate structures from irreversible damage. However, the breakdown of this immune privilege is possible, usually due to host genetic factors and/or environmental factors, and leads to inflammation, as in multiple sclerosis (MS) and autoimmune uveitis. Little is known about the role of IL-24 in the context of autoimmune diseases in CNS. IL-24 gene expression is comparable in the peripheral blood mononuclear cells (PBMCs) of patients with relapsing or stable MS and healthy controls [59]. Interestingly, our recent study revealed that IL-24 deficiency exacerbates both experimental autoimmune encephalomyelitis (EAE) and EAU with elevated pathogenic Th17 response [8]. Our mechanistic studies confirmed that IL-24 directly suppresses the production of proinflammatory cytokines, including IL-17F and GM-CSF, from Th17 cells through the induction of SOCS1 and SOCS3 [8]. This finding suggests a potential role of IL-24 in the protection of the central nervous system from inflammation through the regulation of effector T cells.

### 3.6. Liver Inflammation and Fibrosis

Liver inflammation can be caused by various stimuli, such as viral infection, drugs, alcohol, and genetic/metabolic disorders. The associated excessive tissue damage and inflammatory environment activates monocytes, hepatocytes, Kupffer cells, and stellate cells for the production of proinflammatory molecules and fibrogenic factors [60]. IL-24 was shown to display anti-tumor effects and inhibit hepatocellular carcinoma metastasis [61,62], but its role in regulating liver inflammation and fibrosis is yet to be well characterized. An in vitro study reported that hepatic stellate cells express high levels of IL-24 upon stimulation [63]. However, its expression was reduced in biopsy specimens from patients with severe liver fibrosis [64]. An in vivo study demonstrated that administration of IL-24 significantly reduced liver inflammation and fibrosis after liver injury in mice by inhibiting activation of hepatic stellate cells [64]. Taken together, these studies indicate that IL-24 inhibits hepatic stellate cells in an autocrine manner and limits liver inflammation and fibrosis.

### 3.7. Allergic Diseases

IL-24 is considered a Th2 cytokine, and its role in several allergic diseases—including allergic airway inflammation and allergic skin inflammation—has been well studied. An elevated level of IL-24 was reported in the nasal secretions of patients with allergic rhinitis [65] and was reduced by allergen-specific immunotherapy [66], suggesting an association between IL-24 and the pathogenesis of allergic rhinitis.

IL-24 is a crucial mediator of allergic contact dermatitis. It was upregulated in a mouse model of allergic contact dermatitis induced by 2,4-dinitrofluorobenzene (DNFB) and patients with phenylenediamine-induced contact hypersensitivity [4]. IL-24 is also pathogenic in atopic dermatitis, leading to epithelial barrier dysfunction and *S. aureus* infection. These studies together suggest the critical role of IL-24 in the pathogenesis of allergic skin inflammation.

### 3.8. Therapeutic Potential of Targeting IL-24

Collectively, the current data suggest that IL-24 displays both pro- and anti-inflammatory properties depending on the type of autoimmunity and the site of inflammation. Its pathogenic role in psoriasis and allergic skin inflammation is well demonstrated. The overexpression of IL-24 by genetic modification and the induction of IL-24 by other proinflammatory cytokines, i.e., TNF-α and IL-17A, could exacerbate disease development [44,49]. Thus, the neutralization of IL-24 or blocking of its signaling pathway may lead to a resolution of these skin inflammatory diseases. However, IL-24 seems to play a protective role in other autoimmune and inflammatory diseases—at least in animal studies, such as IBD, MS, uveitis, and liver fibrosis. Further studies are required to better characterize the exact role of IL-24 in these diseases.

## 4. Conclusions

Numerous studies have focused on the pathological role of IL-24 due to the association between its elevation and many inflammatory and autoimmune diseases; however, mechanistic studies have highlighted its potential function in maintaining homeostasis through the regulation of both epithelial and immune cells. As such, greater effort should be made to characterize the complex role of IL-24 in inflammatory and autoimmune diseases. In summary, IL-24 is a multifunctional cytokine; better understanding of its complex regulatory functions is expected to lead to the development of new therapeutic strategies for human diseases.

## Figures and Tables

**Table 1:** The cellular sources of IL-24.

	Cellular Sources	Ref
Immune Cells	T cells	[8,9,10,11,12,13,14]
	B cells	[15]
	NK cells	[16,17]
	Monocytes and Macrophages	[17,18]
Epithelial Cells	Keratinocytes	Reviewed in [3,7]
	Fibroblasts	

**Table 2:** IL-24 in inflammatory and autoimmune diseases.

Diseases	Role
Psoriasis	high expression in skin lesion promotes skin inflammation activates keratinocytes
RA	high expression in inflamed joint promotes mononuclear cell infiltration
IBD	highly expressed in inflamed mucosa protects and maintains epithelial and mucosal integrity
MS and Uveitis	IL-24 deficiency leads to overactivation of Th17 cells and exacerbates both EAE and EAU
Liver inflammation	reduced expression in severe liver fibrosis inhibits hepatic stellate cells and reduces liver inflammation and fibrosis

## References

[1] Huang E.Y., Madireddi M.T., Gopalkrishnan R.V., Leszczyniecka M., Su Z., Lebedeva I.V., Kang D., Jiang H., Lin J.J., Alexandre D. (2001). Genomic structure, chromosomal localization and expression profile of a novel melanoma differentiation associated (mda-7) gene with cancer specific growth suppressing and apoptosis inducing properties. Oncogene.

[2] Fuson K.L., Zheng M., Craxton M., Pataer A., Ramesh R., Chada S., Sutton R.B. (2009). Structural mapping of post-translational modifications in human interleukin-24: Role of N-linked glycosylation and disulfide bonds in secretion and activity. J. Biol. Chem..

[3] Rutz S., Wang X., Ouyang W. (2014). The IL-20 subfamily of cytokines—From host defence to tissue homeostasis. Nat. Rev. Immunol..

[4] Van Belle A.B., Cochez P.M., de Heusch M., Pointner L., Opsomer R., Raynaud P., Achouri Y., Hendrickx E., Cheou P., Warnier G. (2019). IL-24 contributes to skin inflammation in Para-Phenylenediamine-induced contact hypersensitivity. Sci. Rep..

[5] Wang Z., Wang Y., Chen Y., Lv J. (2016). The IL-24 gene protects human umbilical vein endothelial cells against H_2_O_2_-induced injury and may be useful as a treatment for cardiovascular disease. Int. J. Mol. Med..

[6] Imaeda H., Nishida A., Inatomi O., Fujiyama Y., Andoh A. (2011). Expression of interleukin-24 and its receptor in human pancreatic myofibroblasts. Int. J. Mol. Med..

[7] Chen J., Caspi R.R., Chong W.P. (2018). IL-20 receptor cytokines in autoimmune diseases. J. Leukoc. Biol..

[8] Chong W.P., Mattapallil M.J., Raychaudhuri K., Bing S.J., Wu S., Zhong Y., Wang W.W., Chen Z., Silver P.B., Jittayasothorn Y. (2020). The Cytokine IL-17A Limits Th17 Pathogenicity via a Negative Feedback Loop Driven by Autocrine Induction of IL-24. Immunity.

[9] Schaefer G., Venkataraman C., Schindler U. (2001). Cutting edge: FISP (IL-4-induced secreted protein), a novel cytokine-like molecule secreted by Th2 cells. J. Immunol..

[10] Sahoo A., Lee C.G., Jash A., Son J.S., Kim G., Kwon H.K., So J.S., Im S.H. (2011). Stat6 and c-Jun mediate Th2 cell-specific IL-24 gene expression. J. Immunol..

[11] Wei L., Vahedi G., Sun H.W., Watford W.T., Takatori H., Ramos H.L., Takahashi H., Liang J., Gutierrez-Cruz G., Zang C. (2010). Discrete roles of STAT4 and STAT6 transcription factors in tuning epigenetic modifications and transcription during T helper cell differentiation. Immunity.

[12] Anuradha R., George P.J., Hanna L.E., Kumaran P., Chandrasekaran V., Nutman T.B., Babu S. (2014). Expansion of parasite-specific CD4^+^ and CD8^+^ T cells expressing IL-10 superfamily cytokine members and their regulation in human lymphatic filariasis. PLoS Negl. Trop. Dis..

[13] Kumar N.P., Moideen K., Banurekha V.V., Nair D., Babu S. (2018). Modulation of Th1/Tc1 and Th17/Tc17 responses in pulmonary tuberculosis by IL-20 subfamily of cytokines. Cytokine.

[14] Yosef N., Shalek A.K., Gaublomme J.T., Jin H., Lee Y., Awasthi A., Wu C., Karwacz K., Xiao S., Jorgolli M. (2013). Dynamic regulatory network controlling TH17 cell differentiation. Nature.

[15] Maarof G., Bouchet-Delbos L., Gary-Gouy H., Durand-Gasselin I., Krzysiek R., Dalloul A. (2010). Interleukin-24 inhibits the plasma cell differentiation program in human germinal center B cells. Blood.

[16] Poindexter N.J., Walch E.T., Chada S., Grimm E.A. (2005). Cytokine induction of interleukin-24 in human peripheral blood mononuclear cells. J. Leukoc. Biol..

[17] Dabitao D., Hedrich C.M., Wang F., Vacharathit V., Bream J.H. (2018). Cell-Specific Requirements for STAT Proteins and Type I IFN Receptor Signaling Discretely Regulate IL-24 and IL-10 Expression in NK Cells and Macrophages. J. Immunol..

[18] Garn H., Schmidt A., Grau V., Stumpf S., Kaufmann A., Becker M., Gemsa D., Siese A. (2002). IL-24 is expressed by rat and human macrophages. Immunobiology.

[19] Sauane M., Gopalkrishnan R.V., Lebedeva I., Mei M.-X., Sarkar D., Su Z.-Z., Kang D.-C., Dent P., Pestka S., Fisher P.B. (2003). *Mda-7/IL-24* induces apoptosis of diverse cancer cell lines through JAK/STAT-independent pathways. J. Cell Physiol..

[20] Anuradha R., Munisankar S., Dolla C., Kumaran P., Nutman T.B., Babu S. (2016). Modulation of CD4^+^ and CD8^+^ T-Cell Function by Interleukin 19 and Interleukin 24 During Filarial Infections. J. Infect. Dis..

[21] Yang H.L., Zhou W.-J., Lu H., Lei S.-T., Ha S.-Y., Lai Z.-Z., Zheng Z.-M., Ruan L.-Y., He Y.-Y., Li D.-J. (2019). Decidual stromal cells promote the differentiation of CD56(bright) CD16(-) NK cells by secreting IL-24 in early pregnancy. Am. J. Reprod. Immunol..

[22] Rao L.Z., Wang Y., Zhang L., Wu G., Zhang L., Wang F.-X., Chen L.-M., Sun F., Jia S., Zhang S. (2021). IL-24 deficiency protects mice against bleomycin-induced pulmonary fibrosis by repressing IL-4-induced M2 program in macrophages. Cell Death Differ..

[23] Jiang H., Su Z.Z., Lin J.J., Goldstein N.I., Young C.S., Fisher P.B. (1996). The melanoma differentiation associated gene mda-7 suppresses cancer cell growth. Proc. Natl. Acad. Sci. USA.

[24] Emdad L., Bhoopathi P., Talukdar S., Pradhan A.K., Sarkar D., Wang X.-Y., Das S.K., Fisher P.B. (2020). Recent insights into apoptosis and toxic autophagy: The roles of MDA-7/IL-24, a multidimensional anti-cancer therapeutic. Semin. Cancer Biol..

[25] Su Z.Z., Madireddi M.T., Lin J.J., Young C.S.H., Kitada S., Reed J.C., Goldstein N.I., Fisher P.B. (1998). The cancer growth suppressor gene mda-7 selectively induces apoptosis in human breast cancer cells and inhibits tumor growth in nude mice. Proc. Natl. Acad. Sci. USA.

[26] Dash R., Dmitriev I., Su Z.-Z., Bhutia S.K., Azab B., Vozhilla N., Yacoub A., Dent P., Curiel D.T., Sarkar D. (2010). Enhanced delivery of mda-7/IL-24 using a serotype chimeric adenovirus (Ad.5/3) improves therapeutic efficacy in low CAR prostate cancer cells. Cancer Gene Ther..

[27] Ramesh R., Ito I., Saito Y., Wu Z., Mhashikar A.M., Wilson D.R., Branch C.D., Roth J.A., Chada S. (2004). Local and systemic inhibition of lung tumor growth after nanoparticle-mediated mda-7/IL-24 gene delivery. DNA Cell Biol..

[28] Wang C.J., Xue X.B., Yi J.L., Chen K., Zheng J.W., Wang J., Zeng J.P., Xu R.H. (2006). Melanoma differentiation-associated gene-7, MDA-7/IL-24, selectively induces growth suppression, apoptosis in human hepatocellular carcinoma cell line HepG2 by replication-incompetent adenovirus vector. World J. Gastroenterol..

[29] Sarkar D., Su Z.Z., Lebedeva I.V., Sauane M., Gopalkrishnan R.V., Valerie K., Dent P., Fisher P.B. (2002). mda-7 (IL-24) Mediates selective apoptosis in human melanoma cells by inducing the coordinated overexpression of the GADD family of genes by means of p38 MAPK. Proc. Natl. Acad. Sci. USA.

[30] Rahmani M., Mayo M., Dash R., Sokhi U.K., Dmitriev I.P., Sarkar D., Dent P., Curiel D.T., Fisher P.B., Grant S. (2010). Melanoma differentiation associated gene-7/interleukin-24 potently induces apoptosis in human myeloid leukemia cells through a process regulated by endoplasmic reticulum stress. Mol. Pharmacol..

[31] Sauane M., Gopalkrishnan R.V., Choo H.T., Gupta P., Lebedeva I.V., Yacoub A., Dent P., Fisher P.B. (2004). Mechanistic aspects of mda-7/IL-24 cancer cell selectivity analysed via a bacterial fusion protein. Oncogene.

[32] Dent P., Yacoub A., Hamed H.A., Park M.A., Dash R., Bhutia S.K., Sarkar D., Wang X.Y., Gupta P., Emdad L. (2010). The development of MDA-7/IL-24 as a cancer therapeutic. Pharmacol. Ther..

[33] Oral H.B., Kotenko S.V., Yilmaz M., Mani O., Zumkehr J., Blaser K., Akdis C.A., Akdis M. (2006). Regulation of T cells and cytokines by the interleukin-10 (IL-10)-family cytokines IL-19, IL-20, IL-22, IL-24 and IL-26. Eur. J. Immunol..

[34] Zhang Y., Liu Y., Xu Y. (2019). Interleukin-24 Regulates T Cell Activity in Patients with Colorectal Adenocarcinoma. Front. Oncol..

[35] Chung S.H., Ye X.Q., Iwakura Y. (2021). Interleukin 17 family members in health and disease. Int. Immunol..

[36] Hadife N., Nemos C., Frippiat J.P., Hamadé T., Perrot A., Dalloul A. (2013). Interleukin-24 mediates apoptosis in human B-cells through early activation of cell cycle arrest followed by late induction of the mitochondrial apoptosis pathway. Leuk. Lymphoma.

[37] Chong W.P., van Panhuys N., Chen J., Silver P.B., Jittayasothorn Y., Mattapallil M.J., Germain R.N., Caspi R.R. (2015). NK-DC crosstalk controls the autopathogenic Th17 response through an innate IFN-gamma-IL-27 axis. J. Exp. Med..

[38] Mujal A.M., Delconte R.B., Sun J.C. (2021). Natural Killer Cells: From Innate to Adaptive Features. Annu. Rev. Immunol..

[39] Tang Y., Sun X., Wang Y., Luan H., Zhang R., Hu F., Sun X., Li X., Guo J. (2021). Role of IL-24 in NK cell activation and its clinical implication in systemic lupus erythematosus. Clin. Rheumatol..

[40] Yang H.L., Wang C.J., Lai Z.Z., Yang S.L., Zheng Z.M., Shi J.W., Li M.Q., Shao J. (2020). Decidual stromal cells maintain decidual macrophage homeostasis by secreting IL-24 in early pregnancy. Am. J. Reprod. Immunol..

[41] Buzas K., Oppenheim J.J., Zack Howard O.M. (2011). Myeloid cells migrate in response to IL-24. Cytokine.

[42] Li H.H., Lin Y.C., Chen P.J., Hsiao C.H., Lee J.Y., Chen W.C., Tzung T.Y., Wu J.C., Chang M.S. (2005). Interleukin-19 upregulates keratinocyte growth factor and is associated with psoriasis. Br. J. Dermatol..

[43] Bech R., Otkjaer K., Birkelund S., Vorup-Jensen T., Agger R., Johansen C., Iversen L., Kragballe K., Rømer J. (2014). Interleukin 20 protein locates to distinct mononuclear cells in psoriatic skin. Exp. Dermatol..

[44] Kumari S., Bonnet M.C., Ulvmar M.H., Wolk K., Karagianni N., Witte E., Uthoff-Hachenberg C., Renauld J.C., Kollias G., Toftgard R. (2013). Tumor necrosis factor receptor signaling in keratinocytes triggers interleukin-24-dependent psoriasis-like skin inflammation in mice. Immunity.

[45] Lynde C.W., Poulin Y., Vender R., Bourcier M., Khalil S. (2014). Interleukin 17A: Toward a new understanding of psoriasis pathogenesis. J. Am. Acad. Dermatol..

[46] Boehncke W.H., Schon M.P. (2015). Psoriasis. Lancet.

[47] Patel D.D., Kuchroo V.K. (2015). Th17 Cell Pathway in Human Immunity: Lessons from Genetics and Therapeutic Interventions. Immunity.

[48] Krueger J.G., Wharton K.A., Schlitt T., Suprun M., Torene R.I., Jiang X., Wang C.Q., Fuentes-Duculan J., Hartmann N., Peters T. (2019). IL-17A inhibition by secukinumab induces early clinical, histopathologic, and molecular resolution of psoriasis. J. Allergy Clin. Immunol..

[49] Xu X., Prens E., Florencia E., Leenen P., Boon L., Asmawidjaja P., Mus A.M., Lubberts E. (2021). Interleukin-17A Drives IL-19 and IL-24 Expression in Skin Stromal Cells Regulating Keratinocyte Proliferation. Front. Immunol..

[50] Kragstrup T.W., Otkjaer K., Holm C., Jørgensen A., Hokland M., Iversen L., Deleuran B. (2008). The expression of IL-20 and IL-24 and their shared receptors are increased in rheumatoid arthritis and spondyloarthropathy. Cytokine.

[51] Kragstrup T.W., Greisen S.R., Nielsen M.A., Rhodes C., Stengaard-Pedersen K., Hetland M.L., Hørslev-Petersen K., Junker P., Østergaard M., Hvid M. (2016). The interleukin-20 receptor axis in early rheumatoid arthritis: Novel links between disease-associated autoantibodies and radiographic progression. Arthritis Res. Ther..

[52] Liu X., Zhou H., Huang X., Cui J., Long T., Xu Y., Liu H., Yu R., Zhao R., Luo G. (2016). A Broad Blockade of Signaling from the IL-20 Family of Cytokines Potently Attenuates Collagen-Induced Arthritis. J. Immunol..

[53] Li R.C., Guo J., Su L.C., Huang A.F. (2019). Elevated levels of IL-24 in systemic lupus erythematosus patients. Lupus.

[54] Zhang M., Xu W.D., Zhu Y., Wen P.F., Leng R.X., Pan H.F., Ye D.Q. (2014). Serum levels of cytokines in systemic lupus erythematosus: Association study in a Chinese population. Z. Rheumatol..

[55] Andoh A., Shioya M., Nishida A., Bamba S., Tsujikawa T., Kim-Mitsuyama S., Fujiyama Y. (2009). Expression of IL-24, an activator of the JAK1/STAT3/SOCS3 cascade, is enhanced in inflammatory bowel disease. J. Immunol..

[56] Fonseca-Camarillo G., Furuzawa-Carballeda J., Granados J., Yamamoto-Furusho J.K. (2014). Expression of interleukin (IL)-19 and IL-24 in inflammatory bowel disease patients: A cross-sectional study. Clin. Exp. Immunol..

[57] Rokonay R., Veres-Székely A., Szebeni B., Pap D., Lippai R., Béres N.J., Veres G., Szabó A.J., Vannay Á. (2020). Role of IL-24 in the mucosal remodeling of children with coeliac disease. J. Transl. Med..

[58] Onody A., Veres-Székely A., Pap D., Rokonay R., Szebeni B., Sziksz E., Oswald F., Veres G., Cseh Á., Szabó A.J. (2021). Interleukin-24 regulates mucosal remodeling in inflammatory bowel diseases. J. Transl. Med..

[59] Muls N., Nasr Z., Dang H.A., Sindic C., van Pesch V. (2017). IL-22, GM-CSF and IL-17 in peripheral CD4^+^ T cell subpopulations during multiple sclerosis relapses and remission. Impact of corticosteroid therapy. PLoS ONE.

[60] Hernandez-Gea V., Friedman S.L. (2011). Pathogenesis of liver fibrosis. Annu. Rev. Pathol..

[61] Menezes M.E., Bhatia S., Bhoopathi P., Das S.K., Emdad L., Dasgupta S., Dent P., Wang X.Y., Sarkar D., Fisher P.B. (2014). MDA-7/IL-24: Multifunctional cancer killing cytokine. Adv. Exp. Med. Biol..

[62] Ma C., Zhao L.L., Zhao H.J., Cui J.W., Li W., Wang N.Y. (2018). Lentivirusmediated MDA7/IL24 expression inhibits the proliferation of hepatocellular carcinoma cells. Mol. Med. Rep..

[63] Jamhiri I., Hosseini S.Y., Mehrabani D., Khodabandeh Z., Yaghobi R., Dowran R., Zahri S. (2017). The pattern of IL-24/mda-7 and its cognate receptors expression following activation of human hepatic stellate cells. Biomed. Rep..

[64] Wang H.H., Huang J.H., Sue M.H., Ho W.C., Hsu Y.H., Chang K.C., Chang M.S. (2021). Interleukin-24 protects against liver injury in mouse models. EBioMedicine.

[65] Zissler U.M., Chaker A.M., Effner R., Ulrich M., Guerth F., Piontek G., Dietz K., Regn M., Knapp B., Theis F.J. (2016). Interleukin-4 and interferon-gamma orchestrate an epithelial polarization in the airways. Mucosal Immunol..

[66] Zissler U.M., Jakwerth C.A., Guerth F., Lewitan L., Rothkirch S., Davidovic M., Ulrich M., Oelsner M., Garn H., Schmidt-Weber C.B. (2021). Allergen-specific immunotherapy induces the suppressive secretoglobin 1A1 in cells of the lower airways. Allergy.

